# Novel mutations of RPGR in Chinese families with X-linked retinitis pigmentosa

**DOI:** 10.1186/s12886-019-1250-7

**Published:** 2019-11-27

**Authors:** Zhimeng Zhang, Hehua Dai, Lei Wang, Tianchang Tao, Jing Xu, Xiaowei Sun, Liping Yang, Genlin Li

**Affiliations:** 1 Beijing Tongren Eye Center, Beijing Tongren Hospital, Capital Medical University; Beijing Ophthalmology & Visual Sciences Key Lab, Beijing, People’s Republic of China; 2 Department of Ophthalmology, Key Laboratory of Vision Loss and Restoration, Ministry of Education, Peking University Third Hospital, Beijing, People’s Republic of China

**Keywords:** RPGR, X-linked retinitis pigmentosa, ORF15, Nonsense mutation, Pathological myopia

## Abstract

**Background:**

RP (retinitis pigmentosa) is a group of hereditary retinal degenerative diseases. XLRP is a relatively severe subtype of RP. Thus, it is necessary to identify genes and mutations in patients who present with X-linked retinitis pigmentosa.

**Methods:**

Genomic DNA was extracted from peripheral blood. The coding regions and intron-exon boundaries of the retinitis pigmentosa GTPase regulator (RPGR) and RP2 genes were amplified by PCR and then sequenced directly. Ophthalmic examinations were performed to identify affected individuals from two families and to characterize the phenotype of the disease.

**Results:**

Mutation screening demonstrated two novel nonsense mutations (c.1541C > G; p.S514X and c.2833G > T; p.E945X) in the RPGR gene. The clinical manifestation of family 1 with mutations in exon 13 was mild. Genotype-phenotype correlation analysis suggested that patients with mutations close to the downstream region of ORF15 in family 2 manifested an early loss of cone function. Family 2 carried a nonsense mutation in ORF15 that appeared to have a semi-dominant pattern of inheritance. All male patients and two female carriers in family 2 manifested pathological myopia (PM), indicating that there may be a distinctive X-linked genotype-phenotype correlation between RP and PM.

**Conclusions:**

We identified two novel mutations of the RPGR gene, which broadens the spectrum of RPGR mutations and the phenotypic spectrum of the disease in Chinese families.

## Background

Retinitis pigmentosa (RP) is a group of inherited retinal degenerative diseases. The prevalence of RP was estimated to be 1/3000 to 1/4000 in the general population [1, 2]. The symptoms of nyctalopia begin at early onset. With progressive photoreceptor degeneration, patients lose their peripheral visual fields and eventually reach blindness. Ocular examination of RP reveals typical bone-spicule deposits, an attenuation of retinal blood vessels, disc pallor and an opacification of the posterior capsule.

To date, at least 65 different genes have been identified for the autosomal dominant (adRP), autosomal recessive (arRP), and X-linked (XLRP) forms of RP: 22 for adRP, 40 for arRP, and 3 for XLRP (retinitis pigmentosa GTPase regulator [RPGR], RP2 and OFD1). (https://sph.uth.edu/retnet/sum-dis.htm#B-diseases). XLRP accounts for approximately 15% of all RP families [3, 4] and is a relatively severe subtype of RP with onset at early age and rapid progression. Most affected males experience nyctalopia and a restriction of visual fields in the first to second decade of life and a loss central visual acuity in the third decade and reach blindness in their forties [5]. To our knowledge, six loci, RP2, RP3 [RPGR], RP6, RP23, RP24 and RP34, have been identified in XLRP. In addition, RPGR and RP2 gene mutations are important in causing XLRP. Previous studies of XLRP cases have shown that over 70% of mutations in the RPGR gene and approximately 8 to 15% of mutations in the RP2 gene lead to XLRP [3, 6, 7]. There is only one report about a deep intronic mutation in the OFD1 gene (RP23) causing XLRP in one family to date [8].

The RPGR gene is located on Xp21.1, the short arm of the X chromosome [9]. It undergoes complex selective splicing and encodes multiple protein isoforms. There are two widely expressed RPGR isoforms: RPGR^ex 1–19^ (derived from exons 1–19, encoding a protein of 815 amino acids), which is expressed in a wide variety of tissues; and RPGR^ORF15^ (shares exons 1–14 with RPGR^ex 1–19^ and contains ORF15 as its terminal exon, encoding a protein of 1152 amino acids), which is a retina-enriched transcript [10]. The protein of the RPGR gene is predominantly expressed in the connecting cilia of rods and cones, regulating cilia function and promoting protein transport along the photoreceptor cilium [11, 12]. Previous studies suggested that mutations in exons 1–14 account for less than 25% of XLRP [13], while mutations in ORF15 account for over 50–60% of XLRP. The ORF15 from the RPGRORF15 isoform is repeated many times and is full of purine, thus making ORF15 more susceptible to mutation [1, 12].

XLRP represents a character- or genetic-disease-related gene located on the X chromosome, and these genes are recessive and transmit with the behaviour of the X chromosome. Theoretically, female carriers do not show clinical manifestations. However, some female carriers exhibit severe clinical symptoms, which seems to be an autosomal dominant inheritance in the pedigree [14, 15]. Churchill et al. demonstrated that 8.5% of cases with mutations in the XLRP genes RPGR and RP2 were found in families whose initial diagnosis was adRP [16]. The severely affected female carriers make the characteristics of “male to male” transmission inconspicuous, which makes the genetic diagnosis more confusing.

The objective of the current research was to identify the mutated genes in two Chinese RP families and to characterize the phenotypic manifestation associated with the mutation.

## Materials and methods

### Ethics statement

All tests involving DNA of the patients and their families were approved by Peking University Third Hospital Medical Ethics Committee (No. 2012093). Informed consent was signed by all the patients and/or their legal guardians, and this consent procedure was approved by the ethics committees.

### Patients

All patients and normal controls were identified from Beijing Tongren Eye Center and Peking University Eye Center. The diagnosis of RP was as follows: clinical manifestations were nyctalopia since early childhood, progressive peripheral vision loss, and decreasing visual acuity with age. Fundus examination showed waxy pale optic discs, the attenuation of retinal arterioles and scattered bone-spicule pigmentation in the mid-peripheral retina. All patients’ medical and ophthalmic histories were collected, and ophthalmological examinations were performed. One hundred Chinese Han healthy individuals were selected as the control group. All procedures used in the study were consistent with the principles of the Declaration of Helsinki.

### Mutation screening

The experimental protocol is similar to that of our previous study [12]. Peripheral blood samples were collected from patients. The D2492 Blood DNA Maxi Kit was used to extract genomic DNA in standard methods (Omega Bio-Tek Inc., GA, USA). Touchdown PCR was used to perform amplification of exons 1–19, the exon-intron boundaries of RPGR (NG_009553.1; NM_000328) and exons 1–5 of RP2 (NG_009107.1;NM_006915) with high fidelity Taq polymerase (Invitrogen, Grand Island, NY, USA). Its amplification programme consisted of denaturation at 95 °C for 5 min, 35 cycles of amplification (at 95 °C for 30 s, 14 cycles from 64 °C to 57 °C, every cycle decrease 0.5 °C, then 21 cycles at 57 °C for 30 s, and at 72 °C for 40 s), and finally extension at 72 °C for 10 min [17]. An ABI 3130XL genetic analyser (ABI Applied Biosystem, Foster City, CA, USA) was used to sequence the purified PCR products. The following primers were used in PCR analyses: ORF15 5′-CAGAGATCCTATCAGATGACC-3′ (forward), and 5′-TGTCTGACTGGCCATAATCG-3′ (reverse), with a PCR product of 1630 bp. Four reported reverse primers were used to sequence the PCR products [12]. Sequencher (Gene Codes Corporation, Ann Arbor, MI, USA) was used to analyse the sequencing results; the identified mutations were further assessed for segregation in tested family members and 100 controls by sequencing. The primer programme was used to design primer pairs for individual exons (http://www.yeastgenome.org/cgi-bin/web-primer).

## Results

In this study, two mutations in the RPGR gene were identified in two Chinese families. These two mutations were nonsense mutations (c.1541C → G;p.S514X detected in family 1 and c.2833G → T;p.E945X detected in family 2). Both of these mutations are first reported herein.

**Fig. 1:**
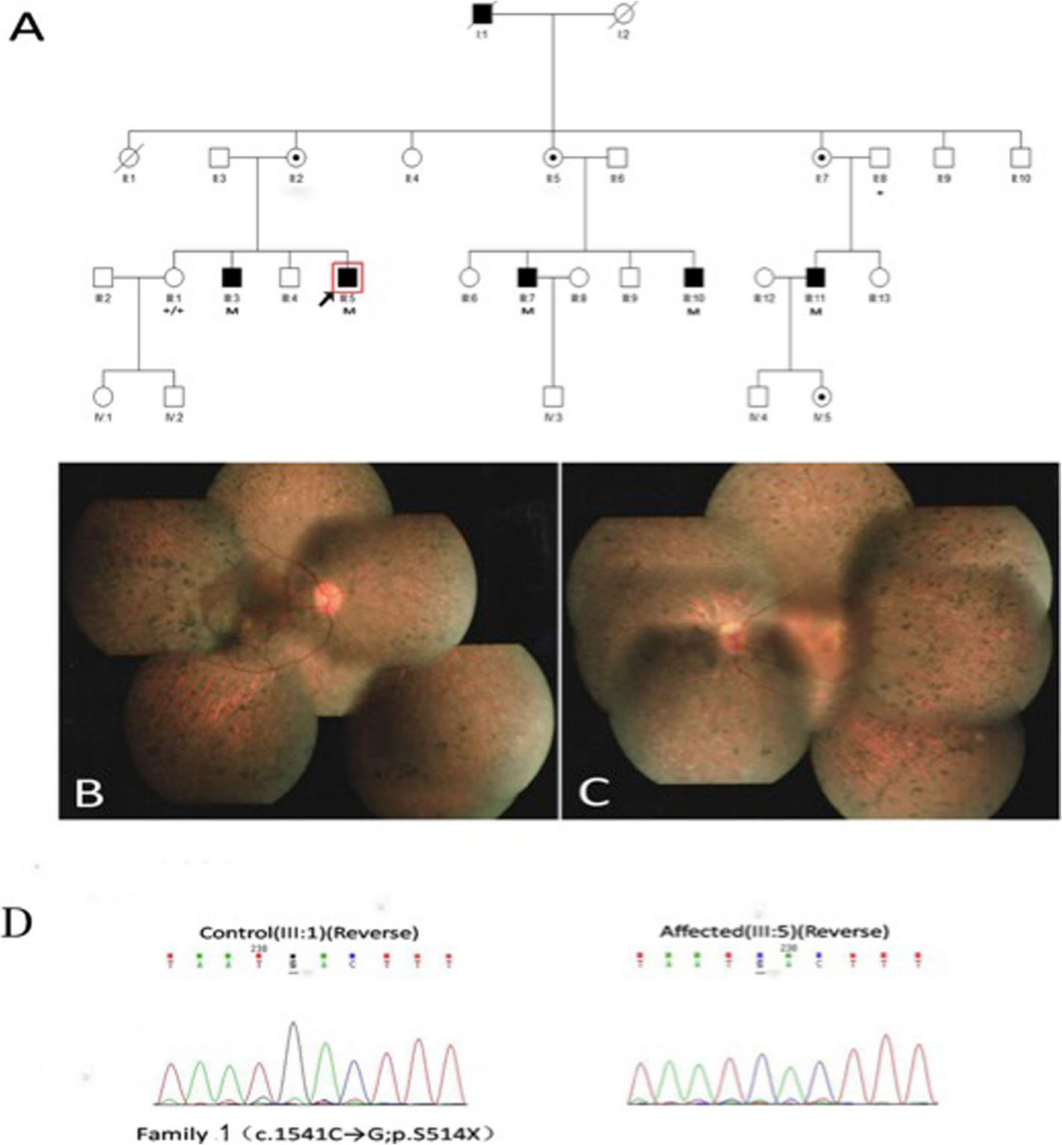
Family 1 with a novel nonsense mutation c.1541C > G(p. S514X) in exon 13 of the RPGR gene. **a** The pedigree of family 1. Filled squares refer to affected patients, unfilled squares or circles represent unaffected individuals, and dotted circles represent carriers. Squares represent males, and circles represent females. Arrow refers to the proband. M marks the mutant allele, and + marks the normal allele. **b**, **c** The 37-year-old proband’s (III:5) fundus photographs showing clinically typical characteristics such as bone-spicule pigment deposits, optic disc pallor, the attenuation of blood vessels and RPE degeneration. **d** The left graph represents a normal sequence chromatogram and the right graph represents a male patient’s sequence chromatogram

In family 1 (Fig. 1a-d), we identified a novel nonsense mutation (c.1541C → G;p.S514X) in exon 13 of RPGR. The 37-year-old proband (III: 5) exhibited typical RP manifestations, for instance, bone-spicule pigment deposition, retinal arteriole attenuation and RPE degeneration (Fig. 1b, c), and had a significant decline in visual acuity in his third decade. His electroretinogram (ERG) indicated the amplitude of rod and cone were reduced (Fig. 3a, b). Female carriers in this family had normal visual function.

**Fig. 2:**
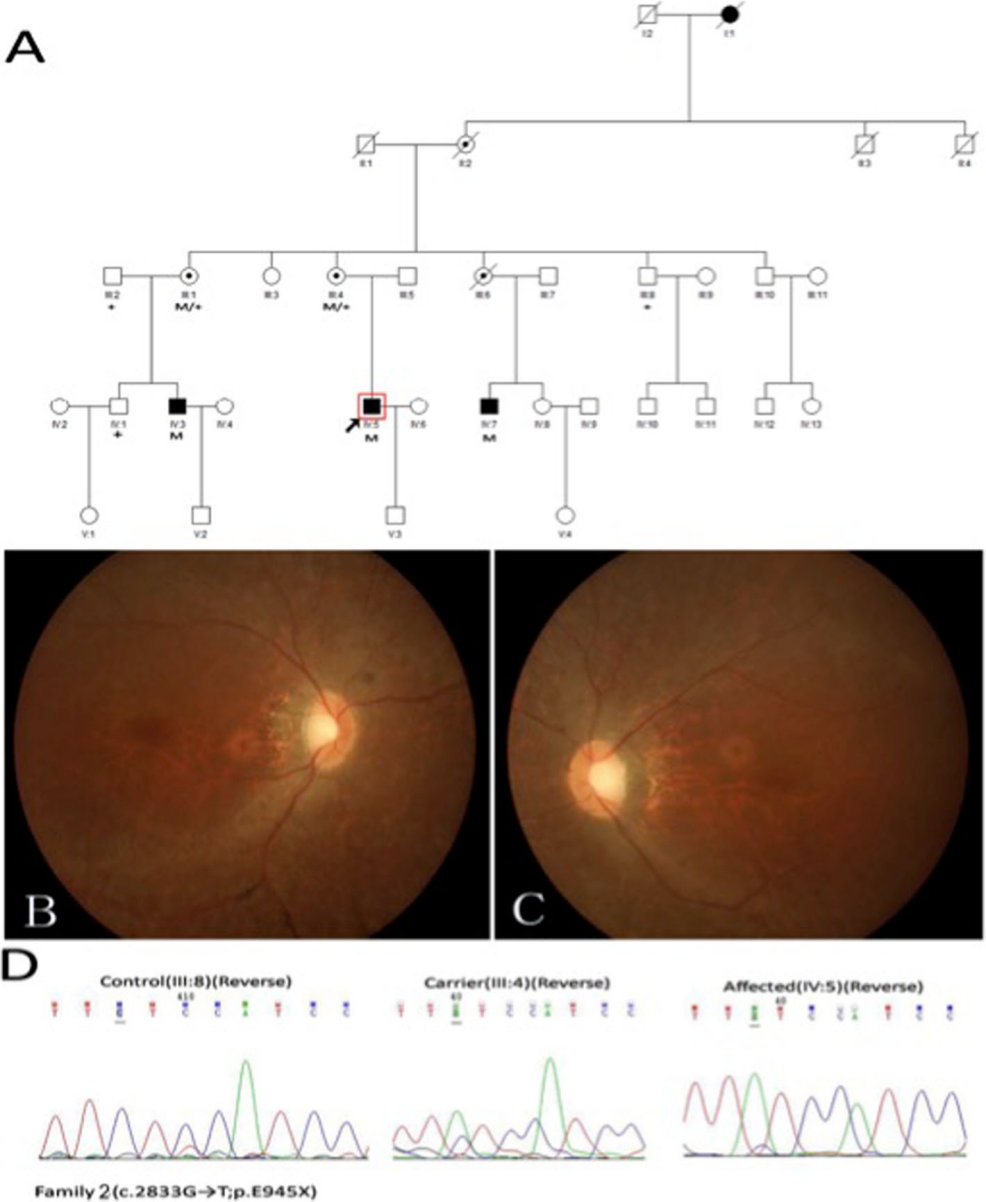
Family 2 with a novel nonsense mutation c.2833G > T(p. E945X) in ORF15 of the RPGR gene. **a** The pedigree of family 2. Filled squares represent to affected patients, unfilled squares or circles represent unaffected individuals, and dotted circles represent carriers. Squares represent males, and circles represent females. Arrow refers to the proband. M marks the mutant allele, and + marks the normal allele. **b**, **c** The 30-year-old proband’s (IV:5) fundus photographs showing pallor of the disc, attenuated blood vessels and tapetal-like fundus change. **d** The left graph represents a normal sequence chromatogram, the middle graph represents a female carrier’s sequence chromatogram and the right graph represents a male patient’s sequence chromatogram

**Fig. 3:**
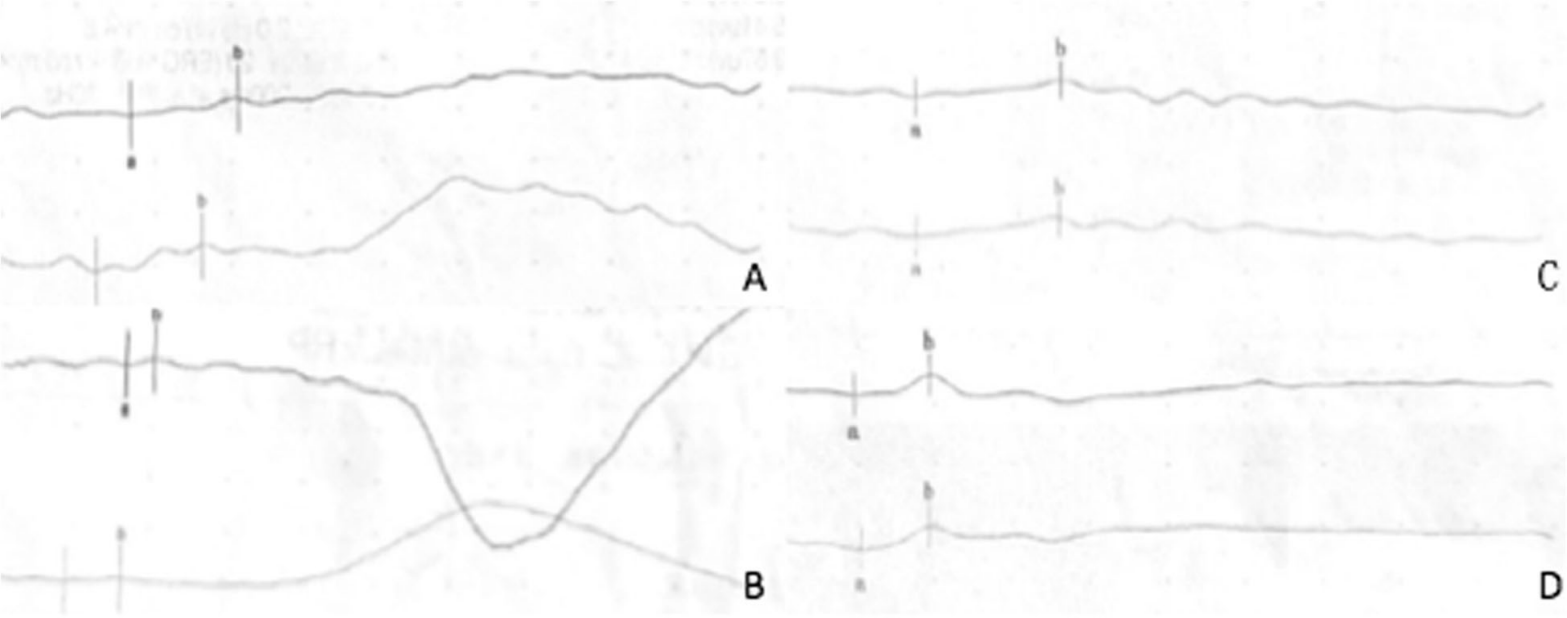
ERG of the probands from the two families. **a** Rod cell response in dark ERG of the proband from family 1. **b** Cone cell response in light ERG of the proband from family 1. **a** and **b** indicated that the amplitude of rod and cone system were reduced, the patient had impaired retinal function in both eyes. **c** Rod cell response in dark ERG of the proband from family 2. **d** Cone cell response in light ERG of the proband from family 2. **c** and **d** indicated that the amplitude of rod and cone system were severely reduced, the patient had severe impaired retinal function in both eyes

In family 2 (Fig. 2a-d), the nonsense mutation (c.2833G → T;p.E945X) happened in ORF15, and the obligatory female carriers manifested varying degrees of fundus phenotypes. A female carrier (III: 4) who was 52 years old had high myopia of approximately -10D to -11D and poor vision at night since childhood. Fundus examinations showed tessellated fundi and a tapetal-like fundus reflex. Another female carrier (III: 6), who is dead, had hypermyopia and severe visual function abnormalities in one eye. However, the fundus examinations of a 60-year-old female carrier (III: 1) showed no obvious abnormal changes, and she had normal visual function. The fundoscopic and functional changes in two female carriers were beyond those in unaffected XLRP carriers; based on this information combined with the onset characteristics of their families, the initial diagnosis of the pedigree was a semi-dominant genetic pattern. The three male patients showed similar fundus changes like the 30-year-old proband (IV: 5) (Fig. 2b, c), which showed pallor of the disc, attenuated blood vessels and tapetal-like fundus change. Figure 3c and d showed the results of his ERG, the amplitude of rod and cone system were severely reduced, the patient had severe impaired retinal function in both eyes. The older patient (IV: 3), who was only 32 years old, was blind (VA: OD: 0.01; OS: hand move (HM)). All of them had high myopia ranging from -6D to -10D.

## Discussion

The RPGR protein is located in the connecting cilium (CC), which is the only junction between the inner segments (IS) and the outer segments (OS) of photoreceptors. Protein transport takes place through the CC. The seven-bladed propeller repeat in the N-terminal part of RPGR, which shows homology to the regulator of chromatin condensation (RCC1), is called RCC1-like domain (RLD) [18]. Thus, RLD is usually regarded as a guanine-nucleotide-exchange factor (GEF) to promote the exchange between GDP and GTP on Ran (Ras-related nuclear protein). RPGR interacts with several partner proteins through RLD, such as RPGRIP1, RPGRIP1L, CEP290, PED6D and RAB8A [19–23], and plays an important role in cilia formation, actin stability and supporting efficient intraflagellar transport (IFT) [24]. Proteins and molecules are transported across the cilium via IFT for normal required OS function, so any mutation in genes encoding proteins covered in IFT can cause a range of different ciliopathies. For example, mutations in RPGRIP1 can cause Leber congenital amaurosis (LCA) and cone-rod dystrophy (CORD), while mutations in RPGRIP1L may cause Joubert syndrome and Meckel syndrome, etc.

Two novel nonsense mutations were detected in the two families. A nonsense mutation means that a triad code that encodes an amino acid is mutated into a termination codon. Although this kind of mutation does not cause errors in the encoding of amino acids, the termination codons then appear in the middle of an mRNA, leading to the premature termination of translation and the formation of an incomplete polypeptide chain. Nguyen LS et al. believed that the premature nonsense mutation before the termination codons may lead to nonsense-mediated decay (NMD) [25, 26]. The NMD of RPGR may occur when frameshift mutations or abnormal stop codons are present in exons 1 to 14. For example, RPGR protein cannot be detected in lymphoblasts of patients with the RPGR Q236X nonsense variant, while both RPGR^ex1–19^ and RPGR^ORF15^ protein isoforms can be detected in the control group [27].. Moreover, Yang L et al. described an NMD (c.851C. G; p. S284X) in exon 8 of RPGR, which was found in a Chinese family with moderate clinical manifestations due to the functional loss of RPGR [12]. ORF15 is the terminal exon of the RPGR^ORF15^ variant transcript. Indeed, the nonsense mutation occurring in ORF15 may lead to stable and abnormal protein production. In general, mutations in exons 1–14, rather than ORF15, are more likely to have severe clinical features in XLRP patients [28], which may be because the truncated protein from the RPGR^ORF15^ variant transcript reserves a normal RLD structure and interacts with other proteins. However, in this study, not only male patients but also female carriers with mutations in ORF15 (family 2) have more severe clinical features than those patients with mutations in exons 1–14 (family 1). This is an interesting phenomenon. Whether it’s because of the mutation in family 1 is in exon 13, which is near the end of the transcript. Although the mutation in exons 1 to 14 led to NMD theoretically, the mutations near the end of the transcript might product truncate protein and the polypeptide chain that is translated is relatively complete. Further experimental and clinical studies are needed to explore the mutation mechanism. Previous studies reported that some mutations in ORF15 also caused cone dystrophy (COD), CORD or macular degeneration [29–31]. Interestingly, all of these mutations were located in the direction of the 3′ end of ORF15, which may produce a slighter protein alteration to maintain better relative rod function [32], while mutations towards the 5′ end of ORF15 were associated with RP in most cases [12, 32]. Talib M et al. believed that the mutations occurring downstream of ORF15 were associated with the preferential loss of cone function rather than the rod function [30]. The older male patients (IV:3) with early-onset visual impairment in family 2 had a rapidly progressed condition and went legally blind at 32 years old while the RP patients went blind at 45 years old in general and the proband (IV:5), who was 29 years old, even had a provisional diagnosis of LCA. These results suggest that patients in family 2 with mutations located in the terminal 3′ region of ORF15 may have early cone involvement, leading to rapid central vision decline.

Female carriers always exhibit some similar symptoms to male subjects, resembling a dominant inheritance pattern [15, 33]. Rozet JM et al. demonstrated that dominant XLRP is usually achieved by truncating the mutation in exon ORF15 of the RPGR gene. The truncated forms of RPGR^ORF15^ might possibly show a dominant, gain-of-function mutation, acting as dominant alleles [34, 35]. Consistent with this finding, the female carriers in family 2 with mutations in ORF15 exhibit a range of phenotypes, varying from asymptomatic to severe retinal disease. However, allelic heterogeneity itself cannot completely explain the observed penetrance of female carriers in families with different mutations of RPGR. For example, Banin E et al. reported an identical missense mutation in exon 8, causing either a recessive or a semi-dominant X-linked pattern of disease in different families [36]. There is not an exact mechanism for this, but the presence of modifier loci that are currently known protein-protein interactions with RPGR or environmental modifiers, or both, may play an important role in severely affected carriers.

In this study, all male patients and two female carriers in family 2 manifested a refractive error ranging from -6D to -11D, indicating that there may be a specific X-linked genotype-phenotype correlation between RP and pathological myopia (PM). According to a co-segregation analysis of a Caucasian family, Parmeggiani F et al. suggested that the PM trait was a possible complete penetrance with all four female carriers heterozygous for ORF15-c.2091_2092insA [37]. Until now, with the exception of the Caucasian family, almost all RP-PM mutations in the mutational hot spot of the RPGR ORF15 gene have been reported in Asian families. With the exception of nonsense mutations in our report, truncating mutations, deletion mutations and frameshift mutations in the exon ORF15 were more frequently reported to lead to the expression of PM [12, 37–39]. The difference between our result and those of previous studies is that not only female carriers have PM but also male patients. Nonsense mutations may also cause this phenomenon. Therefore, PM may be a distinct phenotype of this novel nonsense mutation (c.2833G → T;p.E945X) identified in exon ORF15 of the RPGR gene. In the vertebrate retinas, toxic compounds can be transferred from one cell to another adjacent cell via gap junction channels and/or extracellular routes, after which retinal structure will remodel with photoreceptor degeneration [37, 40]. In this pathogenic context, the mechanism of PM in XLRP patients may be the remodelling of retinal structures via cell-cell degeneration. Furthermore, a recent hypothesis about the relationship between RPGR gene mutations and PM suggested that the location of RPGR protein between IS and OS, was a crucial site affecting vision development [41].

## Conclusions

In conclusion, we found two novel nonsense mutations in two Chinese families with X-linked retinitis pigmentosa and analysed the association between genotypes and phenotypes. We found that there may be a specific X-linked genotype-phenotype correlation between X-linked RP and PM. The findings broaden the genotypic spectrum of RPGR mutations and the phenotypic spectrum of the disease in China. This evidence may be useful for genetic diagnosis or even genetic treatment in the future.

## Data Availability

The datasets generated and analysed during the current study are available from the corresponding author on reasonable request.

## Abbreviations

adRP: Autosomal dominant retinitis pigmentosa
AMD: Atrophic macular degeneration
arRP: Autosomal recessive retinitis pigmentosa
CC: The connecting cilium
COD: Cone dystrophy
CORD: Cone-rod dystrophy
GEF: Guanine-nucleotide-exchange factor
HM: Hand move
IFT: Intraflagellar transport
IS: The inner segments
NMD: Nonsense-mediated decay
OS: The outer segments
PM: Pathological myopia
Ran: Ras-related nuclear protein
RCC1: The regulator of chromatin condensation
RLD: RCC1-like domain
RP: Retinitis pigmentosa
RPGR: Retinitis pigmentosa GTPase regulator
XLRP: X-linked retinitis pigmentosa
